# Profiles and integration of the gut microbiome and fecal metabolites in severe intrahepatic cholestasis of pregnancy

**DOI:** 10.1186/s12866-023-02983-x

**Published:** 2023-10-03

**Authors:** Xiang Li, Han Xie, Jia-jing Chao, Yuan-Hui Jia, Jia Zuo, Yan-peng An, Yi-Rong Bao, Xiang Jiang, Hao Ying

**Affiliations:** 1 Shanghai Key Laboratory of Maternal Fetal Medicine Shanghai Institute of Maternal-Fetal Medicine and Gynecologic Oncology, Shanghai, 200040 China; 2 Department of Obstetrics, Shanghai First Maternity and Infant Hospital, Tongji University School of Medicine, No. 2699, West Gaoke Road, Shanghai, 200040 People’s Republic of China; 3 Clinical and Translational Research Center, Shanghai First Maternity and Infant Hospital, Tongji University School of Medicine, Shanghai, 200040 China; 4 State Key Laboratory of Genetic Engineering, School of Life Sciences, Fudan University, Shanghai, 200438 China

**Keywords:** Intrahepatic cholestasis of pregnancy, Bile acid, Gut microbiome, 16S rRNA, Metabolomics, MIMOSA model

## Abstract

**Background:**

The pathogenesis of intrahepatic cholestasis of pregnancy (ICP) remains unknown. The gut microbiome and its metabolites play important roles in bile acid metabolism, and previous studies have indicated the association of the gut microbiome with ICP.

**Methods:**

We recruited a cohort of 5100 participants, and 20 participants were enrolled in the severe ICP group, matched with 20 participants in the mild ICP group and 20 controls. 16S rRNA sequencing and nontargeting metabolomics were adapted to explore the gut microbiome and fecal metabolites.

**Results:**

An increase in richness and a dramatic deviation in composition were found in the gut microbiome in ICP. Decreased Firmicutes and Bacteroidetes abundances and increased Proteobacteria abundances were found in women with severe but not mild ICP compared to healthy pregnant women. Escherichia-Shigella and Lachnoclostridium abundances increased, whereas Ruminococcaceae abundance decreased in ICP group, especially in severe ICP group. The fecal metabolite composition and diversity presented typical variation in severe ICP. A significant increase in bile acid, formate and succinate levels and a decrease in butyrate and hypoxanthine levels were found in women with severe ICP. The MIMOSA model indicated that genera Ruminococcus gnavus group, Lachnospiraceae FCS020 group, and Lachnospiraceae NK4A136 group contributed significantly to the metabolism of hypoxanthine, which was significantly depleted in subjects with severe ICP. Genus Acinetobacter contributed significantly to formate metabolism, which was significantly enriched in subjects with severe ICP.

**Conclusions:**

Women with severe but not mild ICP harbored a unique gut microbiome and fecal metabolites compared to healthy controls. Based on these profiles, we hypothesized that the gut microbiome was involved in bile acid metabolism through metabolites, affecting ICP pathogenesis and development, especially severe ICP.

**Supplementary Information:**

The online version contains supplementary material available at 10.1186/s12866-023-02983-x.

## Introduction

Intrahepatic cholestasis of pregnancy (ICP) is the most common liver disease unique to pregnancy, with a globally variable prevalence [1–3]. ICP is associated with an increased risk of stillbirth and preterm labor, which cause mortality and morbidity for the newborn [2, 4]. Although studies on cholestasis liver diseases in nonpregnancy have revealed that a number of factors can cause an imbalance in Bile Acids (BAs), including genetics, infections and tumors, none of them can account for the pathogenesis and development of cholestasis in pregnancy [5–8].

The microbial susceptibility hypothesis is supported by the fact that the gut microbiome is involved in BA synthesis and circulation [9–12]. BAs are synthesized from cholesterol by the liver, stored in the gallbladder and metabolized by bacteria in the intestinal tract [13]. Due to the unique tract of the gut-liver axis, the liver is susceptible to the influence of bacterial products from the intestine [14]. Recent studies have found several lines of evidence suggesting that the gut microbiome plays an important role in the etiopathogenesis of cholestatic liver diseases by regulating BA synthesis and metabolism [15, 16]. Experimental and human observational evidence also suggests that the presence and functions of the gut microbiome are relevant for the severity and progression of cholestatic liver disease [17]. Pregnant women harbor a specific gut microbiome composition compared to nonpregnant women [18]. The abundance of Firmicutes and Proteobacteria were increased in pregnant women compared to non-pregnant women. Firmicutes, particularly, were dominant in the second trimester of pregnancy [18]. ICP typically occurs in the late second trimesters when the gut microbiome varies from that in the first trimester of gestation. These facts have led us to hypothesize that an altered gut mycobiome could be essential in the pathogenesis and development of cholestasis in pregnancy. Therefore, analyzing the relationship among the intestinal microbiome, BA metabolism and ICP during pregnancy is of great significance for understanding the onset of ICP.

Recent studies have indicated the association of the gut microbiome with cholestasis in pregnancy, but the results have not been consistent [19–21], which might be the result of heterogeneity among subjects, as studies on other metabolic diseases have also implied the impact of disease severity on the gut microbiome [22]. We found different effects of different severities of ICP on gestational outcomes in our previous study [23]. Therefore, we divided the patients with ICP into different disease severities before further analysis in this study. Furthermore, we explored the causal integration of the gut microbiome and fecal metabolites, providing insights into the possible disease mechanism, which has not been reported in previous studies.

## Materials and methods

### Study participants

We recruited a prospective cohort of 5,100 participants starting from 11 to 13 weeks of gestation at Shanghai First Maternity and Infant Hospital, Tongji University School of Medicine, China, from Jul 2017 to Jul 2018. The inclusion criteria were as follows: singletons; conceived naturally; no infections and had not taken any antibiotics or probiotics for at least 1 month before enrollment. The diagnosis of ICP was based on the following criteria according to the guidelines of the American College of Gastroenterology for ICP [24]: fasting serum total BA (TBA) levels ≥ 10 μmol•L^−1^ and < 40 μmol•L^−1^ for mild ICP and ≥ 40 μmol•L^−1^ for severe ICP. Biological samples were collected at diagnosis and group division, including stool samples and serum samples. The participants were followed up for gestational outcomes and screened and ruled out for any metabolic disorders, other liver diseases or gastrointestinal diseases, infections and other gestational complications or comorbidities until delivery. Eventually, 20 participants were enrolled in the severe ICP group, matched with 20 participants in the mild ICP group and 20 participants in the control group by maternal age, BMI and gestational weeks after excluding cases lost to follow-up. Written informed consent was obtained from each participant when they were recruited. The study was approved by the Ethics Committee of Shanghai First Maternity and Infant Hospital (KS18107) and was carried out according to the reviewed protocol.

### DNA extraction and 16S rRNA sequencing

Total bacterial genomic DNA samples were extracted using Fast DNA SPIN extraction kits (MP Biomedicals, Santa Ana, CA, USA) following the manufacturer’s instructions. PCR amplification of the bacterial 16S rRNA gene V3–V4 region was performed using the forward primer 338F (5’-ACTCCTACGGGAGGCAGCA-3’) and the reverse primer 806R (5’-GGACTACHVGGGTWTCTAAT-3’). PCR amplicons were purified with Agencourt AMPure Beads (Beckman Coulter, Indianapolis, IN) and quantified using a PicoGreen dsDNA Assay Kit (Invitrogen, Carlsbad, CA, USA). After the individual quantification step, the amplicons were pooled in equal amounts, and paired-end 2 × 300 bp sequencing was performed using the Illumina MiSeq platform with MiSeq Reagent Kit v3 at Shanghai Personal Biotechnology Co., Ltd. (Shanghai, China).

### Sequence analysis

The Quantitative Insights Into Microbial Ecology (QIIME, v1.8.0) pipeline was employed to process the sequencing data, as previously described [25]. Valid sequences were identified, and low-quality sequences were filtered. Paired-end reads were assembled. After chimera detection, the remaining high-quality sequences were clustered into operational taxonomic units (OTUs) at 97% sequence identity by UCLUST. A representative sequence was selected from each OTU using default parameters. An OTU table was further generated to record the abundance of each OTU in each sample and the taxonomy of the OTUs. An averaged, rounded rarefied OTU table was generated by averaging 100 evenly resampled OTU subsets under 90% of the minimum sequencing depth for further analysis.

### Metabolites extraction

After the stool samples were thawed at 4 °C, they were shaken and mixed well. One hundred microliters of each sample were transferred to a 1.5 mL EP tube. Precooled 1 mL chloroform/methanol/water (1:2:1, v/v) was added, mixed by vortexing, placed at 4 °C for 2 h, and centrifuged at 13,000 rpm and 4 °C for 15 min. The supernatant was filtered through a 0.22 μm filter membrane, blown dry with nitrogen, and stored at -80 °C for later use. Simultaneously, an equal amount of each sample was collected and mixed into a QC sample following the same procedure above. After reconstitution with 100 μL of polyol/acetonitrile/water (2:5:3 V/V/V), the supernatant was centrifuged for NMR mass spectrometry detection.

### NMR analysis

We have adapted NMR analysis as previous described [26]. All NMR spectra were acquired at 298 K on a Bruker AVANCE III 600 MHz NMR spectrometer (600.13 MHz for 1H frequency) equipped with an inverse cryogenic probe (Bruker Biospin, Germany). For each sample, three spectra were collected, including one with the standard NOESYPR1D sequence (RD − 90° − t1 − 90° − tm − 90° − acq), a t2-edited spectrum with a standard Carr − Purcell − Meibom − Gill (CPMG) sequence (RD − 90° − (τ − 180° − τ)n − acq) with τ of 350 μs and n of 100, and a diffusion-edited spectrum (with the sequence RD − 90° − G1 − τ − 180° − G2 − τ − 90° − Δ − 90° − G3 − τ − 180° − G4 − τ − 90° − te − 90° − acq) with Δ of 200 ms, τ of 150 μs, and te of 5 ms. The 90° pulse length was adjusted to approximately 10 μs for each sample, and 64 transients were collected into 32,000 data points over a spectral width of 20 ppm.

For resonance assignment purposes, a series of two-dimensional (2D) NMR spectra were acquired for selected samples and processed as previously reported. These included 1H − 1H correlation spectroscopy (COSY), 1H − 1H total correlation spectroscopy (TOCSY), J-resolved spectroscopy (JRES), 1H − 13C heteronuclear single quantum correlation (HSQC), and 1H − 13C heteronuclear multiple bond correlation (HMBC) 2D NMR spectra.

### Bioinformatic and statistical analysis

Sequence data analyses were mainly performed using the QIIME and R packages (v3.2.0). (See supplementary materials for details).

## Results

### Demographic and clinical characteristics of subjects

Pregnant women in the three groups were compared according to demographic and clinical characteristics (Table 1). The ages, BMIs before pregnancy, and gestational weeks at enrollment of subjects were not significantly different among the three groups. All participants had no history of cigarette use or alcohol abuse. The TBA level of subjects in the severe ICP group was significantly higher than that in the mild ICP group and in the control group (*p*1 < 0.05, *p*2 < 0.001). The levels of ALT and alkaline phosphatase (ALP) in subjects in the ICP group were significantly higher than those in the control group. The average gestational weeks at delivery of subjects in both ICP groups were significantly lower than those of subjects in the control group. All subjects were singletons, and the average weight of newborns in the severe ICP group was significantly lower than that in the control group (*p* = 0.023).

**Table 1:** Demographic and clinical characteristics of pregnant women enrolled

Variables	Control (*n* = 20)	ICP(Mild, *n* = 20)	ICP(Severe, *n* = 20)	p
Age (year-old)	29.55 ± 3.24	31.10 ± 4.17	32.00 ± 4.05^†^	0.134
BMI before pregnancy (kg•m^−2^)	20.97 ± 2.84	21.16 ± 2.44	21.35 ± 2.44	0.895
Parity				0.551
Primiparous	15(75%)	13(65%)	16(80%)	
Multiparous	5(25%)	7(35%)	4(20%)	
Education Background				0.192
Junior College	1(5%)	1(5%)	2(10%)	
Undergraduate	15(75%)	17(85%)	14(70%)	
Postgraduate	4(20%)	2(10%)	4(20%)	
Gestational Weeks at sampling	36.61 ± 1.50	35.99 ± 2.16	34.44 ± 5.74	0.162
Gestational Weeks at delivery	39.02 ± 1.25	37.49 ± 1.33^#^	36.78 ± 1.55^†^	** < 0.001**
Newborn Weight (g)	3230.50 ± 474.71	2948.25 ± 509.36	2831.75 ± 370.43^†^	**0.023**
TBA(μmol•L^−1^)	3.75 ± 2.40	19.80 ± 8.89^#^	49.35 ± 16.56^†‡^	** < 0.001**
TBIL(μmol•L^−1^)	7.64 ± 2.26	8.12 ± 3.05	9.23 ± 4.33	0.308
DBIL(μmol•L^−1^)	2.05 ± 0.50	2.40 ± 0.84	2.15 ± 2.25	0.462
ALT (U•L^−1^)	10.00 (7.00–14.75)	19.00 (10.00–102.25)^#^	10.00 (7.00–173.25)	**0.042**
AST (U•L^−1^)	15.00 (12.25–18.00)	22.00 (13.25–47.50)	16.50 (12.00–96.50)	0.130
ALP (U•L^−1^)	135.00 (105.50–164.00)	159.00 (121.50–228.00)	122.00 (86.50–149.00)^‡^	**0.036**
γ-GT (U•L^−1^)	8.00 (5.25–23.25)	23.00 (8.00–54.25)	10.50 (9.00–14.75)	0.122
LDH (U•L^−1^)	178.25 ± 41.81	185.30 ± 33.29	194.50 ± 46.22	0.455
Albumin (g•L^−1^)	38.95 ± 3.55	34.02 ± 2.97	34.47 ± 2.97^†^	** < 0.001**
Albumin/Globulin	1.43 ± 0.18	1.29 ± 0.18^#^	1.37 ± 0.19	0.051

Normally distributed continuous variables are shown as means with standard deviations. Variables with significant deviation from normal distribution are shown as median and quartiles. Frequencies with percentages are used to present categorical variables

^#^means significant difference between Control group and Mild ICP group (*p* < 0.05)

^†^means significant difference between Control group and Severe ICP group (*p* < 0.05)

^‡^means significant difference between Mild ICP group and Severe ICP group (*p* < 0.05); Placenta Ratio refers to ratio of Placenta Weight over Newborn Weight

### Diversity and microbial features at the phylum level of the gut microbiome of pregnant women with mild and severe ICP

**Fig. 1:**
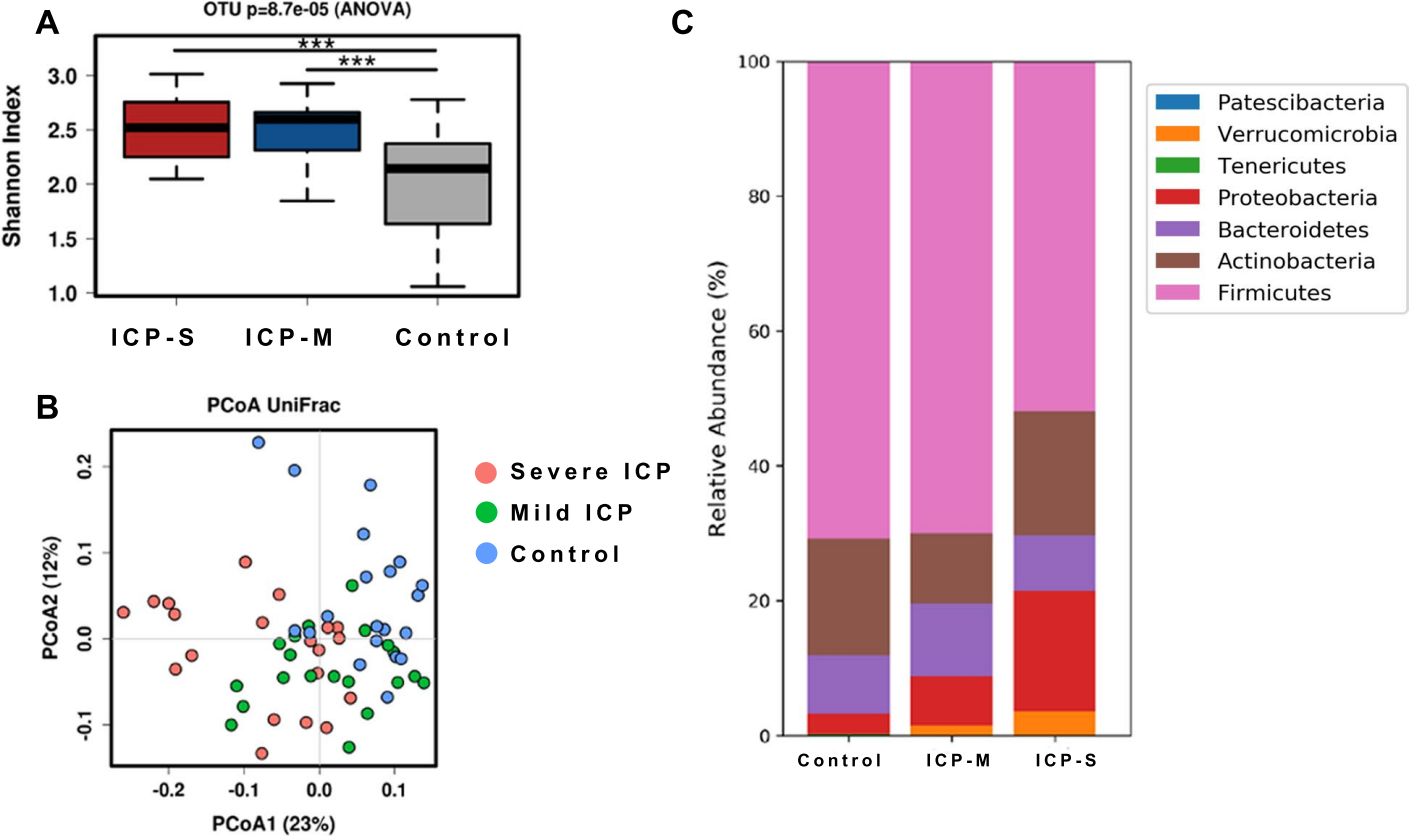
Diversity and microbial features at the phylum level of the gut microbiome of pregnant women with ICP. **A** α diversity of the gut microbiome of subjects in the control, mild ICP and severe ICP groups expressed by the Shannon index. *** indicates a significant difference between groups (*p* < 0.001). **B** β diversity of the gut microbiome of subjects in the control, mild ICP and severe ICP groups expressed according to PCoA based on UniFrac distance. **C** Microbiome composition in different groups at the phylum level. The five most dominant phyla were Firmicutes, Bacteroidetes, Actinobacteria, Proteobacteria and Verrucomicrobia

The richness of the gut microbiome measured by the numbers of observed OTUs was increased in subjects in the severe ICP group and mild ICP group compared with that in the control group (*p* = 8.7 × 10^–5^). Similarly, the Shannon index, which indicates richness and evenness, of the severe ICP group and mild ICP group was significantly higher than that of the control group (*p*_1_ < 0.0001, *p*_2_ < 0.0001) (Fig. 1A). An unweighted UniFrac-based PCoA revealed that the overall composition of the gut microbiome of subjects in the ICP groups deviated dramatically from that of subjects in the control group (Adonis/PERMANOVA *R* = 0.779, *p* = 0.001) (Fig. 1B). The distance between groups was significantly higher than that within groups (*p* = 0.003), which revealed the differences among the three groups. Furthermore, we found that the difference between the severe ICP group and the control group and the difference between the severe ICP group and the mild ICP group were significantly higher than those between the mild ICP group and the control group (Figure S1 A).

For global insight, we first investigated the specific changes in the gut microbiome by assessing the abundance of taxa at the phylum level. Overall, Firmicutes and Proteobacteria were dominant in all subjects, and the mild ICP group and control group harbored a similar phenotype, distinguished to that of the severe ICP group (Fig. 1C). Patescibacteria, Verrucomicrobia, Tenericutes and other phylum were also detected in these samples but the abundances were at very low levels. The abundances of Firmicutes and Bacteroidetes were significantly decreased in subjects in the severe ICP group (Figure S1 C-D). The abundance of Proteobacteria was significantly increased in subjects in the severe ICP group (Figure S1 E). The abundances of Actinobacteria and Verrucomicrobia were not significantly different between the mild or severe ICP and control groups (Figure S1 B, F).

### Microbial features at the genus level of pregnant women with mild and severe ICP

We then explored potential differences in the gut microbiome at the genus level and found that 33 bacterial taxa displayed different abundances among the severe ICP group, mild ICP group, and control group (p_fdr_ < 0.05, Kruskal test).(Table S1) The mild ICP group and control group harbored a similar phenotype, distinguished to that of the severe CP group at the genus level. The abundance of Coprococcus 1, Roseburia, Ruminococcus 1, Tyzzerella 3, Eubacterium eligens group and Eubacterium ventriosum group were found significantly decreased to an extremely low level in severe ICP group compared to control and mild ICP group. (Figure S2 A-F).

**Fig. 2:**
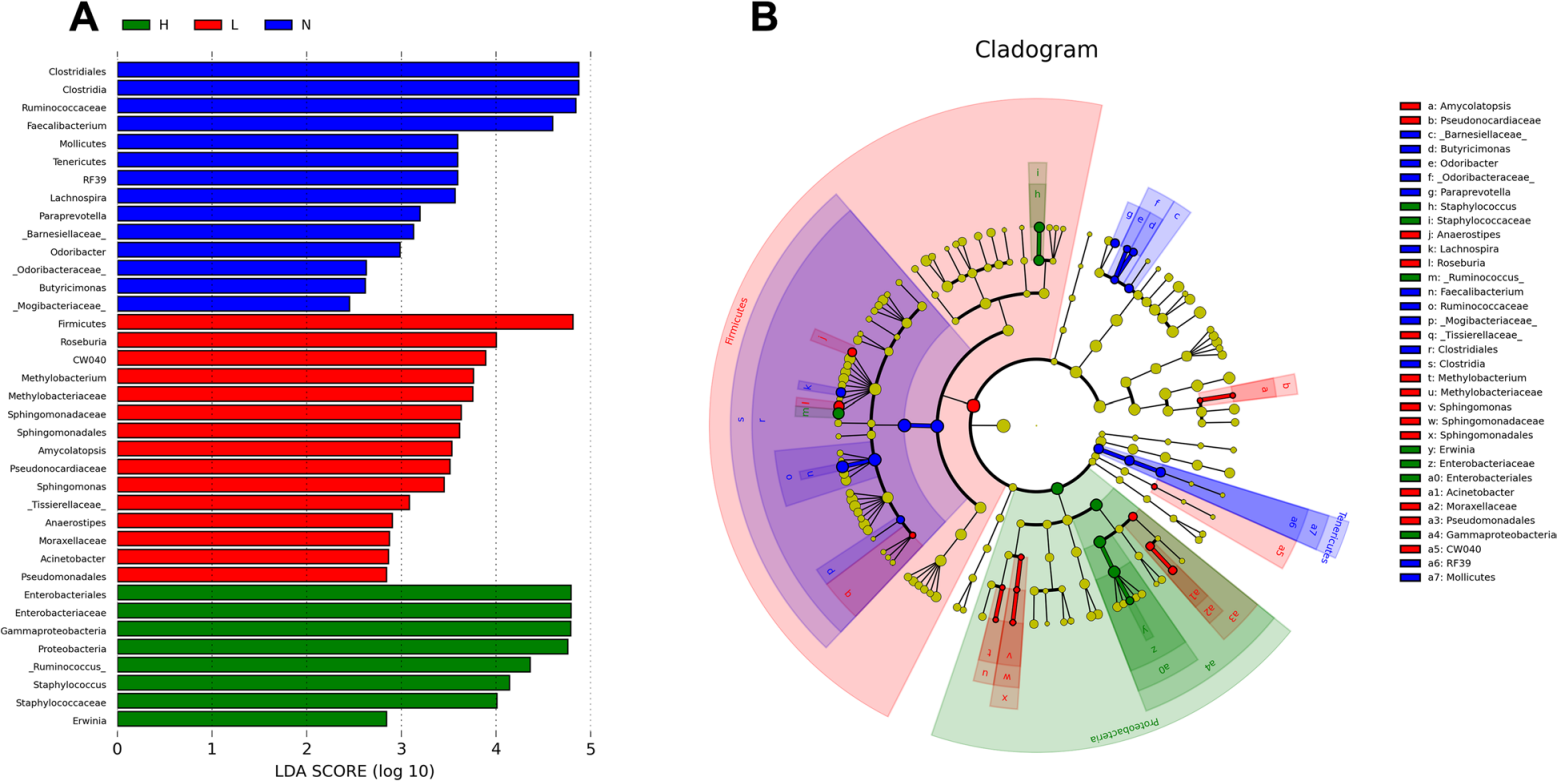
Microbiome composition in different groups at the genus level. **A** Log10 of the LDA score of different genera of the gut microbiome of pregnant women in the control, mild ICP and severe ICP groups; blue bars show severe ICP cases, red bars show mild ICP cases, and green bars show control cases. **B** Evolutionary relationship of differentially abundant gut microbiome constituents at all levels for all enrolled pregnant women (from phylum to genus)

We further applied LEfSe to more accurately reveal the difference in abundance by linear discriminant analysis (LDA) (log10 score > 2) (Fig. 2A). Taxonavigation indicated that most of the differentially abundant genera were from the phyla Firmicutes and Proteobacteria. However, they exhibited substantial variability at the levels from class to family (Fig. 2B).

**Fig. 3:**
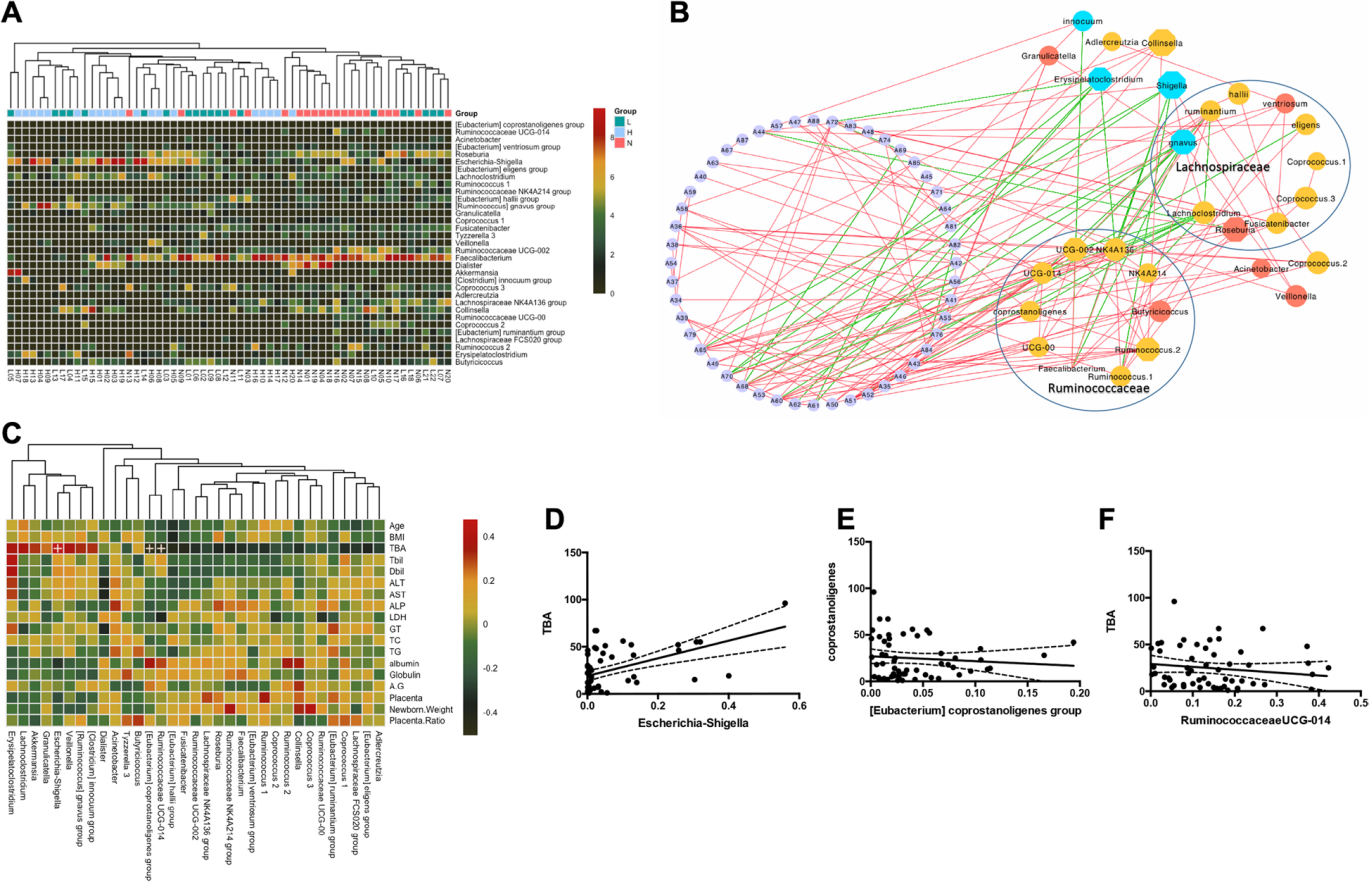
Microbial features at the genus level of pregnant women with ICP. **A** Heatmap of the relative abundance of gut microbiome constituents at the genus level of pregnant women in the control, mild ICP and severe ICP groups (clustered by group, H stands for severe ICP group, L for mild ICP group and N for control group): The community composition of differentially abundant genera was clustered according to the abundance distribution of taxa or the degree of similarity between samples. The taxa and samples were sorted separately according to the clustering results. Through clustering, high- and low-abundance taxa were distinguished, and the color gradient reflects the similarity of community composition between samples. **B** Association of differentially abundant gut microbiome constituents at the genus level for all enrolled pregnant women (clustered by family): A Spearman correlation test was performed to evaluate the relationships among differentially abundant genera among the groups. Red lines show positive associations, and green lines show negative associations. **C** Association of clinical characteristics and differentially abundant gut microbiome constituents at the genus level for all enrolled pregnant women. **D**-**F** Regression analysis of TBA levels and the abundances of Escherichia-Shigella, Ruminococcaceae UCG-014 and (Eubacterium) coprostanoligenes groups

Subjects in all groups displayed a relatively low abundance of differentially abundant taxa overall. Most of the differentially abundant taxa revealed a pattern of all-or-none between subjects with and without ICP (Fig. 3A). Significant positive intragroup correlations were found among taxa in the same group, whereas significant negative intergroup correlations were found among taxa observed from the different groups. Intriguingly, two families of bacteria, Ruminococcaceae and Lachnospiraceae, were the best taxa, with a strongly positive intragroup correlation and a strongly negative intergroup correlation (Fig. 3B). Furthermore, a Spearman correlation test revealed that the TBA level was significantly associated with the abundance of three genera (Fig. 3C). The abundances of Ruminococcaceae UCG-014 and the (Eubacterium) coprostanoligenes group were negatively associated with TBA levels (R1 = -0.68, *p* = 0.021; R2 = -0.69, *p* = 0.017), whereas the abundance of Escherichia-Shigella was positively associated with TBA levels (*R* = 0.57, *p* = 0.014) (Fig. 3D-F).

### Composition and diversity of metabolites in the stool of pregnant women with mild and severe ICP

**Fig. 4:**
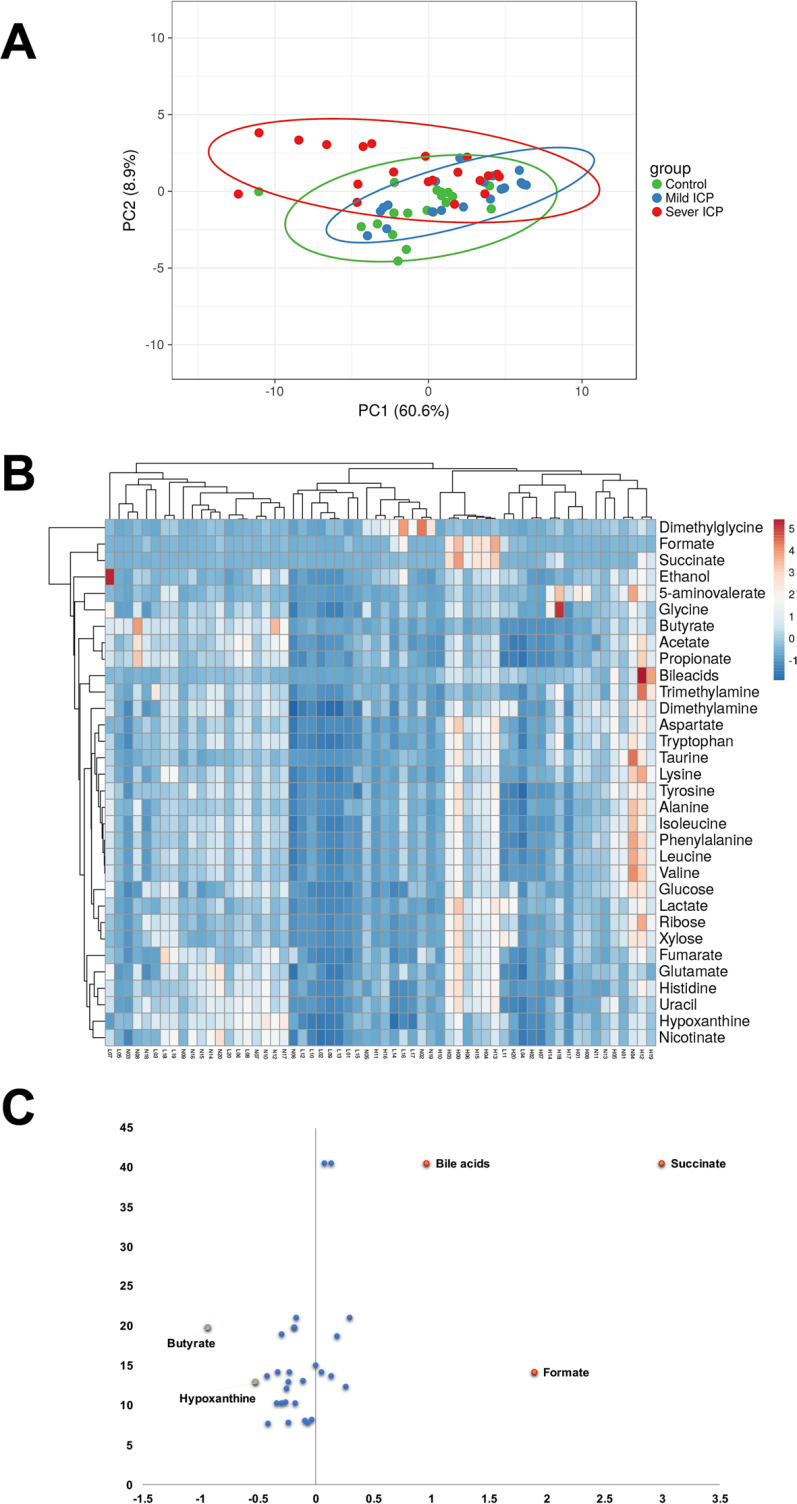
Composition and diversity of metabolites in the stool of women with and without ICP. **A** PCA of metabolites in stool samples of pregnant women in the control, mild ICP and severe ICP groups. **B** Heatmap of the relative abundances of metabolites in stool samples of pregnant women in the control, mild ICP and severe ICP groups (clustered by both group and metabolites, H stands for severe ICP group, L for mild ICP group and N for control group). **C** Volcano chart of fold changes in metabolites in stool samples of pregnant women in the control and ICP groups; red dots show enriched metabolites, and green dots show depleted metabolites

We performed untargeted metabolomics analyses of the stool content of pregnant women with and without ICP. PCA analysis was adapted in order to illustrate compositions of all metabolites among groups. (Fig. 4A) Heatmap illustrated results of cluster analysis of all metabolites among groups, in order to further explore distinguished clusters in severe ICP group. (Fig. 4B) Pregnant women in the severe ICP group harbored a more complicated stool metabolite composition than those in the control group, while pregnant women in the mild ICP group shared a much more similar stool metabolite composition to those in the control group. Pregnant women in the severe ICP group harbored a number of richer and higher-concentration metabolites in stool.

Notably, 5 out of 33 detected metabolites were found to be significantly different among groups (fold change > 0.5, p_fdr_ < 0.05). (Fig. 4C) BA, formate and succinate levels were increased, whereas butyrate and hypoxanthine levels were decreased in subjects in the severe ICP group (Figure S3).

### Association between the microbiome and metabolites in pregnant women with mild and severe ICP

We found that multiple KEGG categories were disturbed in ICP subjects (Figure S4). Strikingly, K15874, K15873, K15872, K15871, K15870, K15869, K15868, K01442, and K00076 and other BA biosynthesis pathways were the most correlated with the gut microbiome (Table S2). There was a negative association between these key KEGG orthologs and the abundances of Ruminococcaceae and Lachnoclostridium, which supported the findings of the composition analysis.

**Fig. 5:**
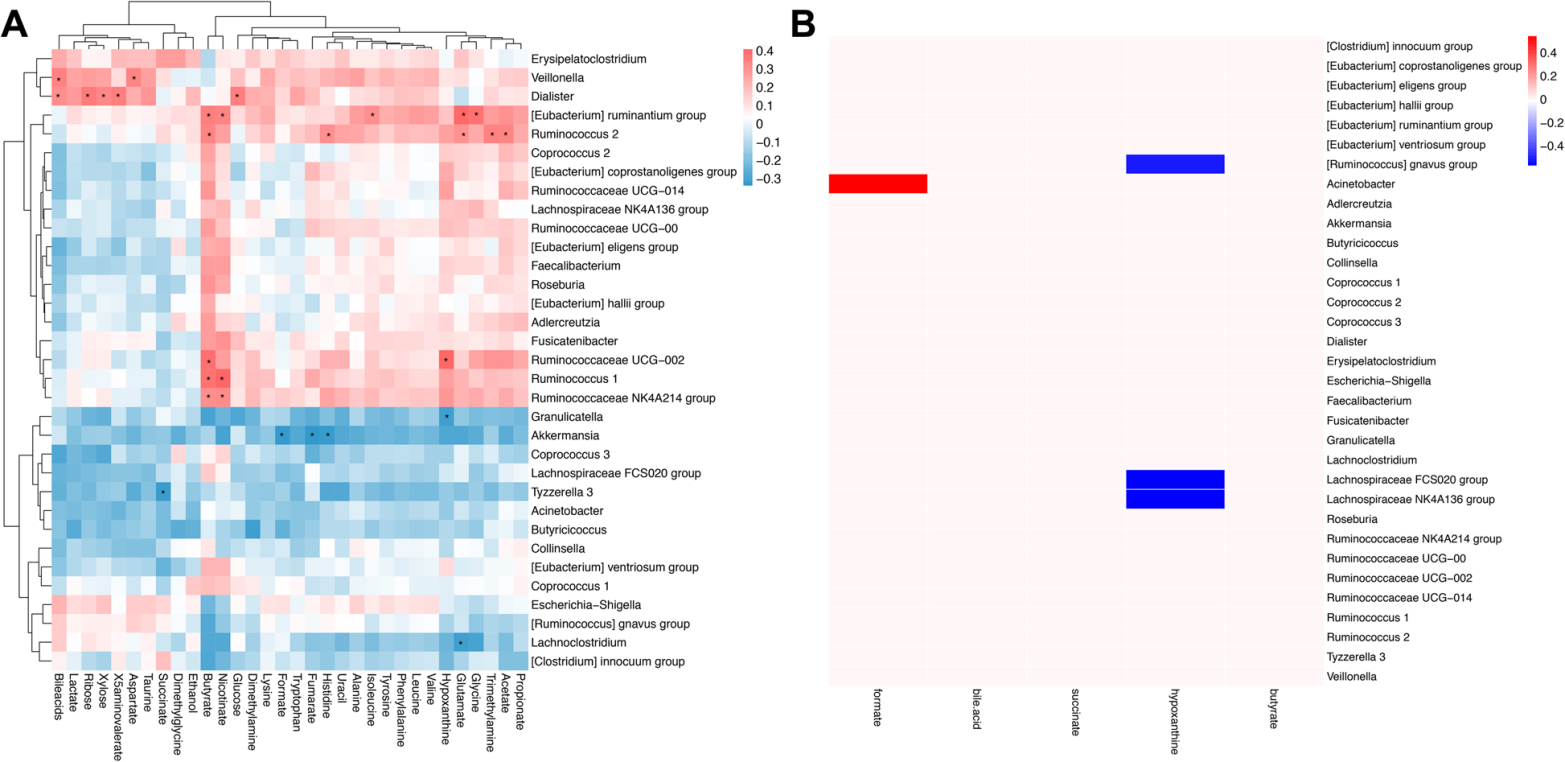
Association of the gut microbiome with fecal metabolites in women with ICP. **A** Heatmap of Spearman's rank correlation coefficients of differentially abundant genera and differentially abundant metabolites in the stool of pregnant women with ICP. **B** MIMOSA model of differentially abundant genera and differentially abundant metabolites in the stool of pregnant women with ICP. Red blocks show enriched metabolites, and blue blocks show depleted metabolites in pregnant women with ICP

We then investigated the correlation between key metabolites in stool and differentially abundant genera of the gut microbiome by the Spearman correlation test (Fig. 5A). Butyrate and hypoxanthine were found to be associated with several differentially abundant genera, including Ruminococcaceae and Ruminococcus.

Finally, we analyzed 16S rRNA data from the microbiome and data from untargeted metabolomics together, coupling them with the MIMOSA [27] model, and investigated the association of key metabolites of differentially abundant genera of the gut microbiome. We found that two metabolites in the ICP group were well predicted. The genera Ruminococcus gnavus group, Lachnospiraceae FCS020 group, and Lachnospiraceae NK4A136 group contributed significantly to the metabolism of hypoxanthine, which was significantly depleted in subjects with severe ICP. The genus Acinetobacter contributed significantly to the metabolism of formate, which was significantly enriched in subjects with severe ICP (Fig. 5B).

## Discussion

### Principal findings

The results reflected increased species abundance as well as a significant shift in the overall microbial diversity in pregnant women with ICP. Interestingly, dysbiosis with higher diversity was observed in patients with severe ICP. Patients with mild ICP shared a gut microbiome similar in features to that of the healthy controls. This result suggested that pregnant women with severe ICP tended to present a strong association of dysbiosis with the disease. Women with severe but not mild ICP harbored a unique gut microbiome and fecal metabolites compared to healthy controls. In addition, causality for the microbiome and metabolite data was provided. The gut microbiome could be involved by affecting metabolites, leading to dynamic BA uptake, efflux, biosynthesis homeostasis and ICP severity.

### Results in the context of what is known

#### Gut microbial features of ICP

In previous studies without classification of the severity of ICP, it was found that the abundance of Firmicutes was decreased, while the abundance of Bacteroidetes was increased at the phylum level; and Enterobacteriaceae, Streptococcaceae, Streptococcus, and Escherichia Shigella abundances increased at the genus level [21]. However, the phenotype of decreased Firmicutes and Bacteroidetes abundances with increased Proteobacteria abundance and increased abundances of Enterobacteriaceae, Gammaproteobacteria, Proteobacteria, Ruminococcus, Enterobacteriales, Erwinia, Staphylococcus and Staphylococcaceae were found only in pregnant women with severe ICP but not in those with mild ICP in our study. We also found that some genera were depleted only in pregnant women with severe ICP but not in those with mild ICP. All these results illustrate that pregnant women with severe ICP exhibit a distinct gut microbiome profile compared to that of healthy controls, while pregnant women with mild ICP share similar gut microbiome features with healthy controls.

When we investigated the correlation between differentially abundant bacteria, the result was that most of them were from the phyla Firmicutes and Proteobacteria. Variable genes among individuals were considered to be associated with Proteobacteria, which indicated the essential role of Proteobacteria in shaping the phenotypes of the gut microbiome [28]. Proteobacterial expansion is a microbial signature of colonic epithelial dysfunction [29]. Pathogenic bacteria can take advantage of this dysfunction and invade the bile ducts. An animal model also confirmed that intestinal microbiome-macrophage crosstalk contributed to cholestatic liver disease by promoting intestinal permeability [30]. Therefore, the identical feature of the gut microbiome in pregnant women with severe ICP could be the result of increased Proteobacteria abundance, which was lacking in pregnant women with mild ICP. Firmicutes is usually known as a protective factor against inflammatory diseases, including nonalcoholic fatty liver disease [31], because Firmicutes bacteria have been reported to be able to ferment carbohydrates to a variety of SCFAs, including butyrate, which is considered a protective factor against inflammation [32]. Considering the mechanical cholestasis caused by inflammation, Firmicutes might also help reduce inflammation in the pathogenesis of ICP. Firmicutes has also been reported to be responsible for the synthesis of tauroursodeoxycholic acid, a secondary BA, from primary BAs in a mouse model [33]. Consequently, decreased Firmicutes abundance could cause cholestasis by directly reducing the metabolism of BAs.

At the genus level, Escherichia-Shigella was increased in abundance in the ICP group, especially in the severe ICP group, and there was a significantly positive association of Escherichia-Shigella abundance with TBA levels. Previous studies have shown that Shigella forms biofilms in the presence of bile salts. The primary bile salt chenodeoxycholate promotes exopolysaccharide production, which supports biofilm formation [34, 35]. This result suggested that there might be positive feedback between Escherichia-Shigella and cholestasis: a high level of BAs in cholestasis facilitates the expansion of Escherichia-Shigella, while Escherichia-Shigella promotes cholestasis, aggravating the disease. Ruminococcaceae abundance was decreased in the ICP group, especially in the severe ICP group, and there was a significantly negative association of Ruminococcaceae abundance with TBA levels. Ruminococcaceae is usually known as a good bacterium for its protective role in liver diseases. Researchers have found that patients with nonalcoholic fatty liver disease have a lower abundance of Oscillibacter (Ruminococcaceae) [36]. Ruminococcaceae has also been reported to be depleted in cirrhosis dysbiosis and associated with a higher end-stage liver disease score [37, 38]. A decreased abundance of Ruminococcaceae might be involved in the pathogenesis of cholestasis.

#### Fecal metabolite features of ICP

Hepatobiliary balance is thought to be directly influenced by microbe-derived cytoprotective and cytodestructive molecules [39]. We predicted the functional composition profiles with PICRUSt and showed that KEGG orthologs of BA biosynthesis were the most correlated with the gut microbiome. In addition, correlations between orthologs and abundant bacteria were intriguingly opposite in subjects in the ICP groups and the control group. This result suggested that these molecules might be the key to disease development pathways.

We then investigated the profile of stool metabolites. Similar to the profile of the gut microbiome, pregnant women with severe ICP harbored distinct stool metabolite features compared to those of the healthy controls, while the pregnant women with mild ICP shared a very similar stool metabolite profile with healthy controls. BAs, formate and succinate were enriched, whereas butyrate and hypoxanthine were depleted in subjects in the severe ICP group. Serum formate levels have been found to be significantly positively correlated with higher sum steatosis scores and elevated ALT and AST levels [40]. Therefore, formate could be responsible for the typical pathophysiological elevation in liver enzyme levels in ICP. Butyrate is usually known as a beneficial factor, helping control inflammation and even preventing disease. It acts as the energy source for intestinal epithelial cells and can reduce the pH in the colon, inhibiting the growth of harmful bacteria. It can also regulate the host's intestinal immunity and reduce inflammation of the colon. Supplementation with butyrate-producing bacteria has been shown to relieve Crohn’s disease by enhancing intestinal epithelial barrier integrity [41].

#### Metabolic model-based framework for comprehensive insights into the gut microbiome and metabolites

In a previous study, a global correlation analysis of the gut microbiome and metabolites was applied, and the results indicated an association of the gut microbiome with BA metabolism [21]. To address how the gut microbiome and microbe-associated molecules interact, we adapted correlation analysis methods, including simple Spearman correlation analysis and the MIMOSA model. A global analysis using Spearman correlation found that Dialister, *Eubacterium ruminantium* group and Ruminococcaceae were the genera that were highly associated with metabolites. Butyrate and hypoxanthine were found to be highly associated with the gut microbiome. As discussed above, bacteria from the family Ruminococcaceae and butyrate could play a protective role in the pathogenesis and development of ICP. The correlation analysis further suggested that Ruminococcaceae was involved in regulating butyrate levels.

Global analysis could not provide causality for the microbiome and metabolite data. Consequently, we applied 16S rRNA sequencing together with untargeted metabolomics coupled with the MIMOSA framework in the analysis of fecal samples to infer the contribution of bacterial species and genes to the production and degradation of metabolites measured by NMR analysis. We found by adapting the MIMOSA model that the genera [Ruminococcus] gnavus group, Lachnospiraceae FCS020 group, and Lachnospiraceae NK4A136 group contributed significantly to hypoxanthine metabolism, which decreased in pregnant women with severe ICP. In the correlation analysis of differentially abundant genera, the family Lachnospiraceae was found to be strongly correlated with other genera. Therefore, the gut microbiome could be involved by affecting Lachnospiraceae, leading to a decrease in the levels of hypoxanthine and preventing its protective effects. In addition, the gut microbiome is able to regulate BA metabolism by reducing the levels of primary BAs and natural farnesoid X receptor (FXR) antagonists [42–45]. Microbiota-induced secondary BAs can act as high-affinity ligand agonists of TGR5, inhibit cytokine secretion and reduce inflammation [46–48]. Consequently, when Firmicutes, which plays a key role in the synthesis of secondary BAs, was depleted in pregnant women with severe ICP, the protective effect mediated by TGR5 and FXR declined.

### Clinical implications

All the findings of our study help illustrate the role of the gut microbiome in the development of ICP and the identification of the severity of the disease. The gut microbiota performs many important functions in the host, establishing a dynamic state of symbiosis. Studies on the mechanism of how the gut microbiome is involved in the pathogenesis of ICP are needed in the future to provide a full understanding and treatment strategies for clinical practice.

### Research implications

We posited that ICP was caused as a consequence of dysbiosis. Moreover, dysbiosis worsened as BA metabolism accelerated, producing a vicious cycle. Therefore, the microbial and metabolic profiles identified in pregnant women with severe ICP may be a potentially powerful tool for disease prediction, and further integrating genetic markers and profiles from multiomics approaches could improve the discriminative ability. A multiomics approach using the MIMOSA model ensures the logic of the evidence and provides a prospective approach for further studies on the mechanism of disease.

### Strengths and limitations

In this study, we indicated the association of the gut microbiome with ICP severity. Furthermore, we explored the causal integration of the gut microbiome and fecal metabolites, providing insights into the possible disease mechanism, which has not been reported in previous studies.

Although our investigations attempted to provide comprehensive insight into the potential contribution of the gut microbiome in the development of ICP, there are several limitations to be addressed in future studies. First, well-characterized chronological cohorts are needed, which can enable the identification of subsets of ICP individuals. Second, the range of gestational weeks at every time point of sample collection should be limited within 4 weeks or less to ensure less intragroup and within-sample diversity. Finally, compared with the limited nature of 16S rRNA sequencing, shotgun sequencing for metagenomics or metatranscriptomics could reveal more accurate information on microbial composition and function.

## Conclusion

Women with severe ICP, not mild ICP, harbored a unique gut microbiome and fecal metabolites compared to those of healthy controls. Based on the profiles of the gut microbiome and fecal metabolites, we hypothesized that the gut microbiome was involved in the metabolism of BAs through metabolites, leading to severe ICP by affecting the development of cholestasis. All these findings help to further understand the pathogenesis of ICP, providing new insight for treatment regimens in clinical practice.

### Supplementary Information

**Additional file 1: Supplementary Method.** Bioinformatic and statistical analysis

**Additional file file 2: Figure S1.** Diversity and microbial features at the phylum level of the gut microbiome of pregnant women with ICP

**Additional file file 3: Figure S2.** Microbiome composition in different groups at the genus level

**Additional file file 4: Figure S3.** Composition and diversity of metabolites in the stool of women with and without ICP

**Additional file file 5: Figure S4.** Heatmap for KEGG Orthologies distribution in sample from different group

**Additional file file 6: Table S1.** Differential bacteria at genus level among groups of control, mild ICP and severe ICP

**Additional file file 7: Table S2.** Key differential KEGG Orthologies among groups of control, mild ICP and severe ICP

## Data Availability

The datasets used and/or analysed during the current study available in the NCBI and Github repository. https://www.ncbi.nlm.nih.gov/bioproject/PRJNA1005000 https://github.com/stephenying2011/ICP2023
