# Supplementary material for: Profiles and integration of the gut microbiome and fecal metabolites in severe intrahepatic cholestasis of pregnancy
Source: BMC Microbiol. 2023 Oct 3;23:282. doi: 10.1186/s12866-023-02983-x (PMC10546765; doi:10.1186/s12866-023-02983-x)
Supplement: Supplementary file 1 — Additional file 1: Supplementary Method. Bioinformatic and statistical analysis [file 12866_2023_2983_MOESM1_ESM.docx]

**Supplementary Method: Bioinformatic and statistical analysis**

OTU-level ranked abundance curves were generated to compare the richness and evenness of OTUs among samples. β diversity analysis was performed to investigate the structural variation in microbial communities across samples using UniFrac distance metrics and visualized via principal coordinate analysis (PCoA), nonmetric multidimensional scaling (NMDS) and unweighted pair-group method with arithmetic means (UPGMA) hierarchical clustering. Differences in the UniFrac distances for pairwise comparisons among groups were determined using Student’s t test and the Monte Carlo permutation test with 1000 permutations. Principal component analysis (PCA) was also conducted based on the genus-level compositional profiles. The significance of microbiota structure differentiation among groups was assessed by permutational multivariate analysis of variance (PERMANOVA) and analysis of similarities (ANOSIM) using the R package “vegan”. The taxonomy compositions and abundances were visualized using MEGAN, and a GraPhlAn Venn diagram was generated to visualize the shared and unique OTUs among samples or groups using the R package “VennDiagram”, based on the occurrence of OTUs across samples/groups regardless of their relative abundances. Taxon abundances at the phylum, class, order, family, genus and species levels were statistically compared among samples or groups by Metastats and visualized as violin plots. Linear discriminant analysis effect size (LEfSe) was performed to detect differentially abundant taxa across groups using the default parameters. Partial least squares discriminant analysis (PLS-DA) was also applied as a supervised model using the “plsda” function in the R package “mixOmics” to reveal the microbiota variation among groups. Random forest analysis was applied to discriminate the samples from different groups using the R package “randomForest” with 1000 trees and all default settings. The generalization error was estimated using 10-fold cross-validation. The expected “baseline” error was also included, which was obtained by a classifier that simply predicts the most common category label. Cooccurrence analysis was performed by calculating Spearman’s rank correlations between predominant taxa. Correlations with |RHO| > 0.6 and P < 0.01 were visualized as cooccurrence networks using Cytoscape. Microbial functions were predicted by Phylogenetic Investigation of Communities by Reconstruction of Unobserved States (PICRUST) based on high-quality sequences.
