# Supplementary material for: Profiles and integration of the gut microbiome and fecal metabolites in severe intrahepatic cholestasis of pregnancy
Source: BMC Microbiol. 2023 Oct 3;23:282. doi: 10.1186/s12866-023-02983-x (PMC10546765; doi:10.1186/s12866-023-02983-x)
Supplement: Supplementary file 2 — Additional file file 2: Figure S1. Diversity and microbial features at the phylum level of the gut microbiome of pregnant women with ICP [file 12866_2023_2983_MOESM2_ESM.pdf]

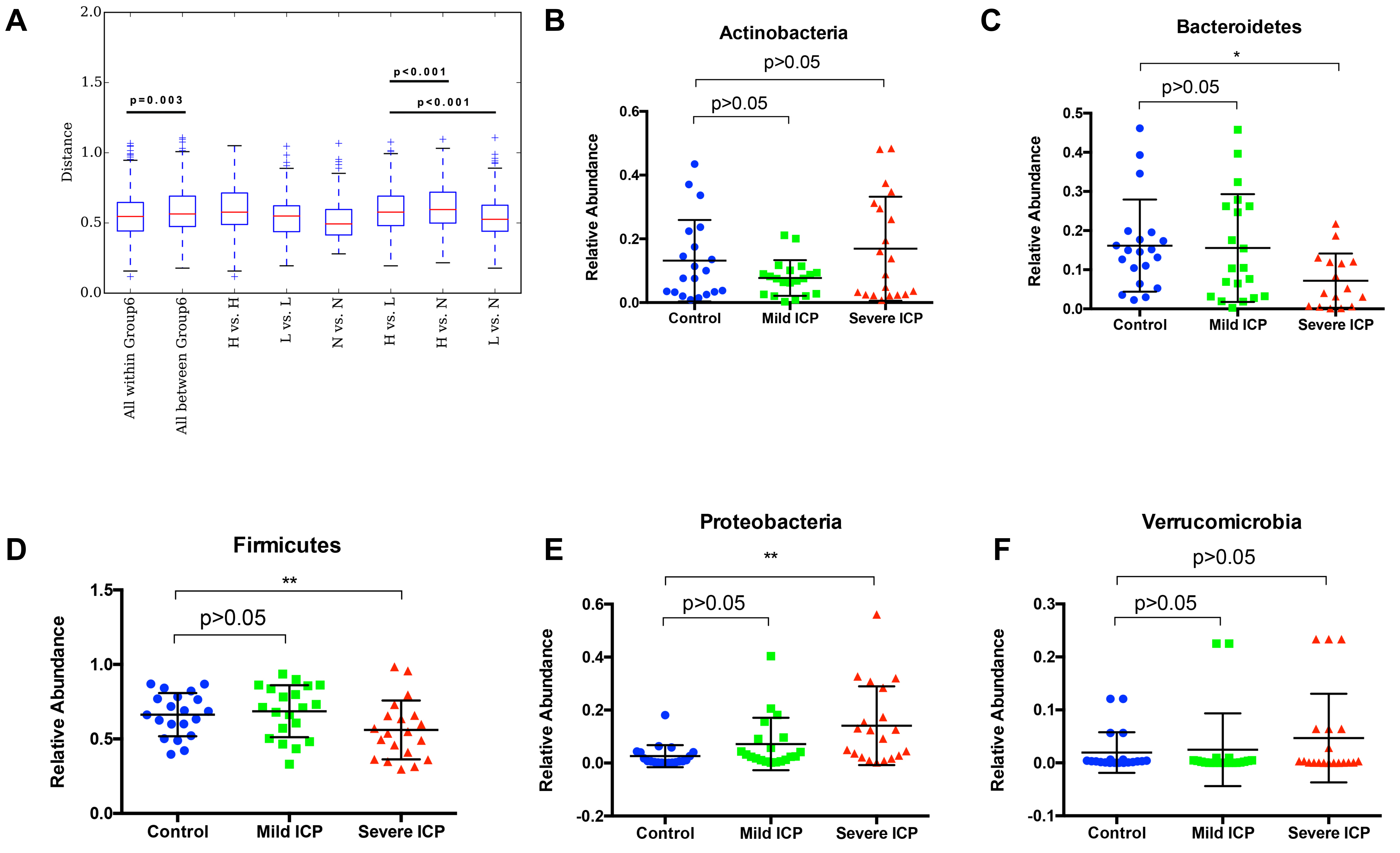

Figure S1. Diversity and microbial features at the phylum level of the gut microbiome of pregnant women with ICP

(A) Unweighted UniFrac-based distance among groups; N indicates the control group, L indicates the mild ICP group, and H indicates the severe ICP group. (B-F) Abundance of representative differentially abundant phyla in different groups: Actinobacteria, Bacteroidetes, Firmicutes, Proteobacteria and Verrucomicrobia.
