# Supplementary material for: Profiles and integration of the gut microbiome and fecal metabolites in severe intrahepatic cholestasis of pregnancy
Source: BMC Microbiol. 2023 Oct 3;23:282. doi: 10.1186/s12866-023-02983-x (PMC10546765; doi:10.1186/s12866-023-02983-x)
Supplement: Supplementary file 3 — Additional file file 3: Figure S2. Microbiome composition in different groups at the genus level [file 12866_2023_2983_MOESM3_ESM.pdf]

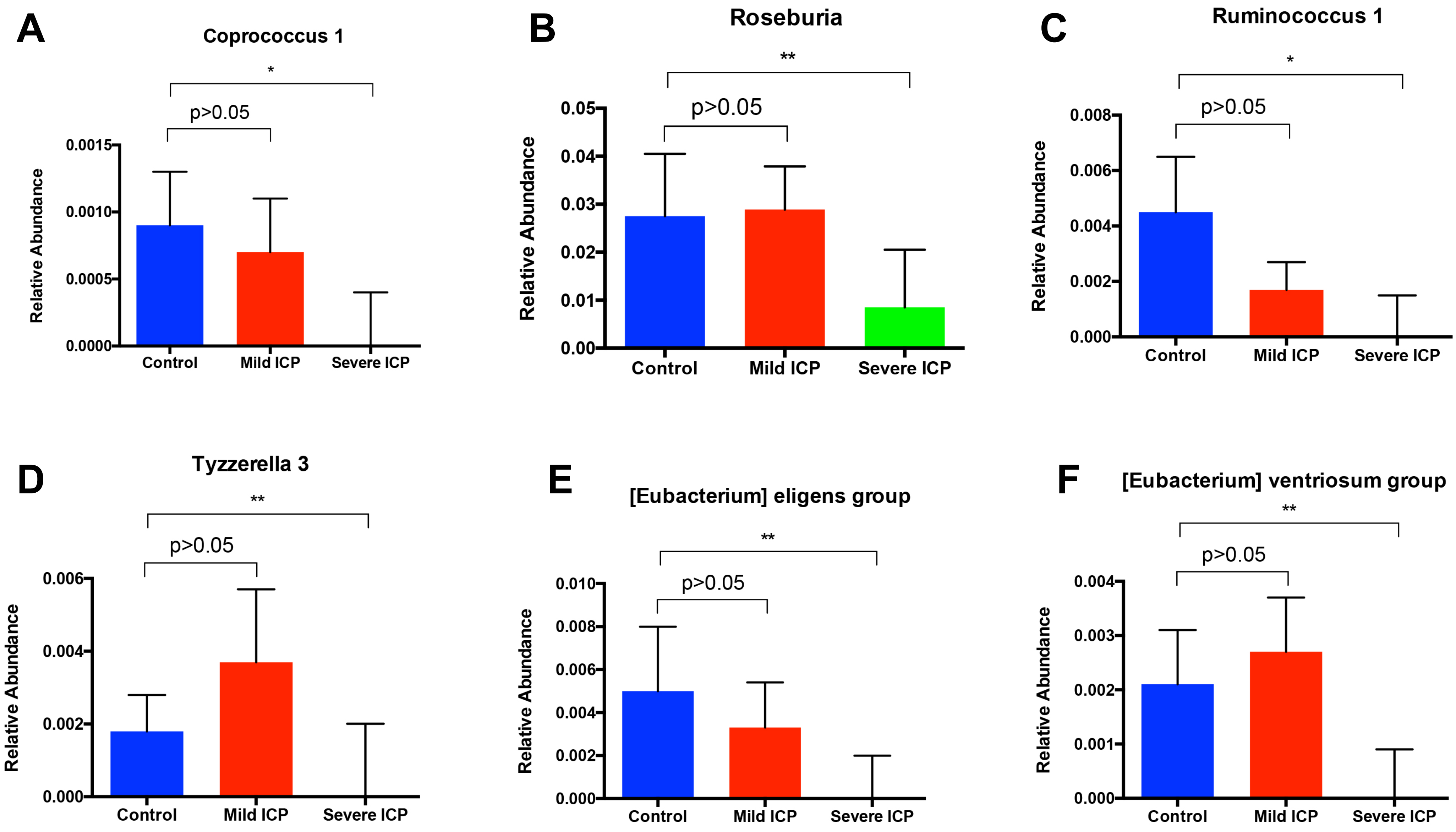

Figure S2. Microbiome composition in different groups at the genus level

(A-F) Abundance of representative differentially abundant genera in the different groups: Coprococcus 1, Roseburia, Ruminococcus 1, Tyzzerella 3, [Eubacterium] eligens group, and [Eubacterium] ventriosum group.
