# Supplementary material for: Profiles and integration of the gut microbiome and fecal metabolites in severe intrahepatic cholestasis of pregnancy
Source: BMC Microbiol. 2023 Oct 3;23:282. doi: 10.1186/s12866-023-02983-x (PMC10546765; doi:10.1186/s12866-023-02983-x)
Supplement: Supplementary file 4 — Additional file file 4: Figure S3. Composition and diversity of metabolites in the stool of women with and without ICP [file 12866_2023_2983_MOESM4_ESM.pdf]

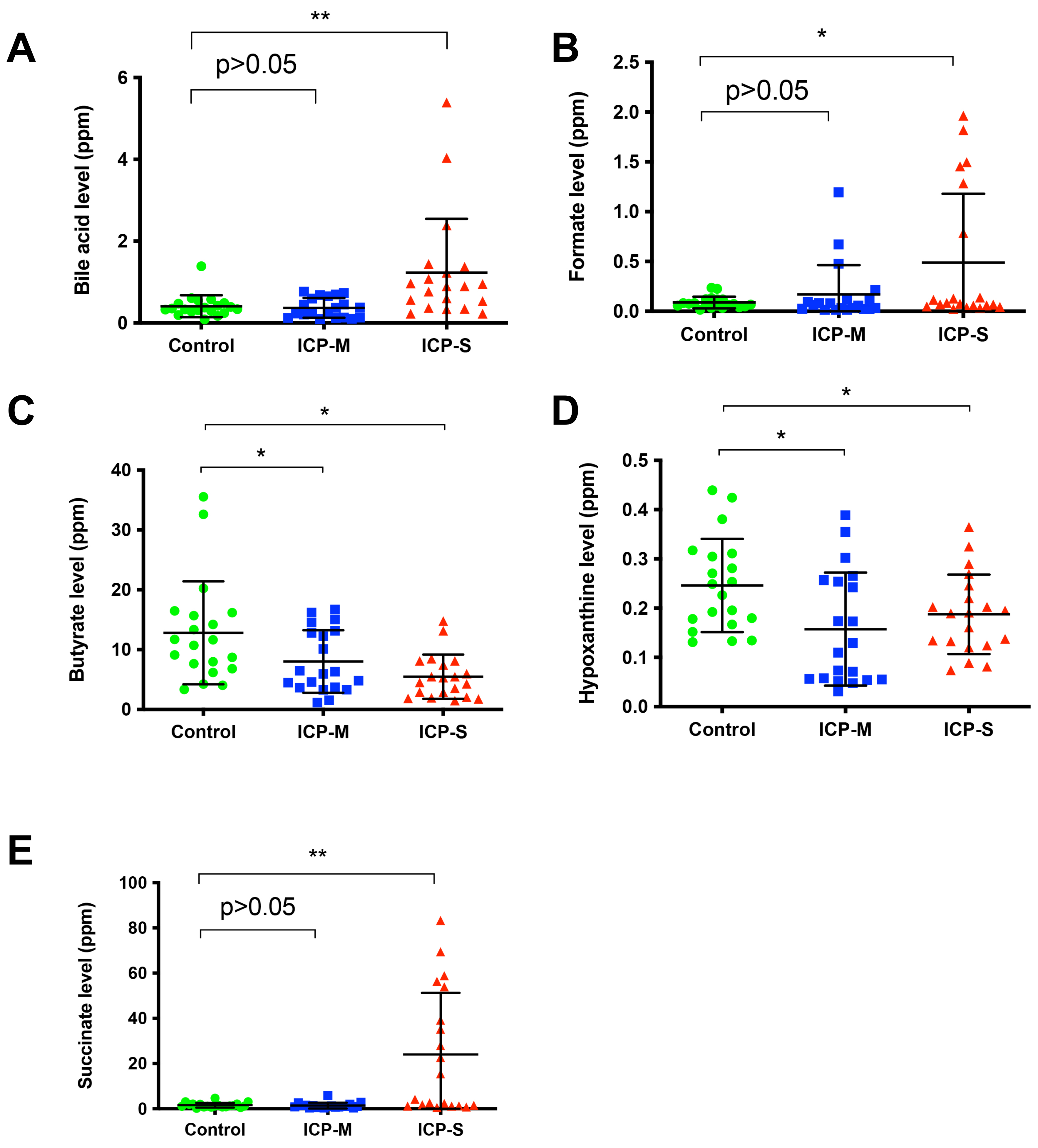

Figure S3. Composition and diversity of metabolites in the stool of women with and without ICP

(A-E) Different levels of metabolites in stool samples of pregnant women in the control, mild ICP and severe ICP groups: D: butyrate, E: hypoxanthine, F: BAs, G: formate, H: succinate. * means $p<0.05$ (t test), ** means $p<0.01$ (t test).
