# Supplementary material for: Profiles and integration of the gut microbiome and fecal metabolites in severe intrahepatic cholestasis of pregnancy
Source: BMC Microbiol. 2023 Oct 3;23:282. doi: 10.1186/s12866-023-02983-x (PMC10546765; doi:10.1186/s12866-023-02983-x)
Supplement: Supplementary file 5 — Additional file file 5: Figure S4. Heatmap for KEGG Orthologies distribution in sample from different group [file 12866_2023_2983_MOESM5_ESM.pdf]

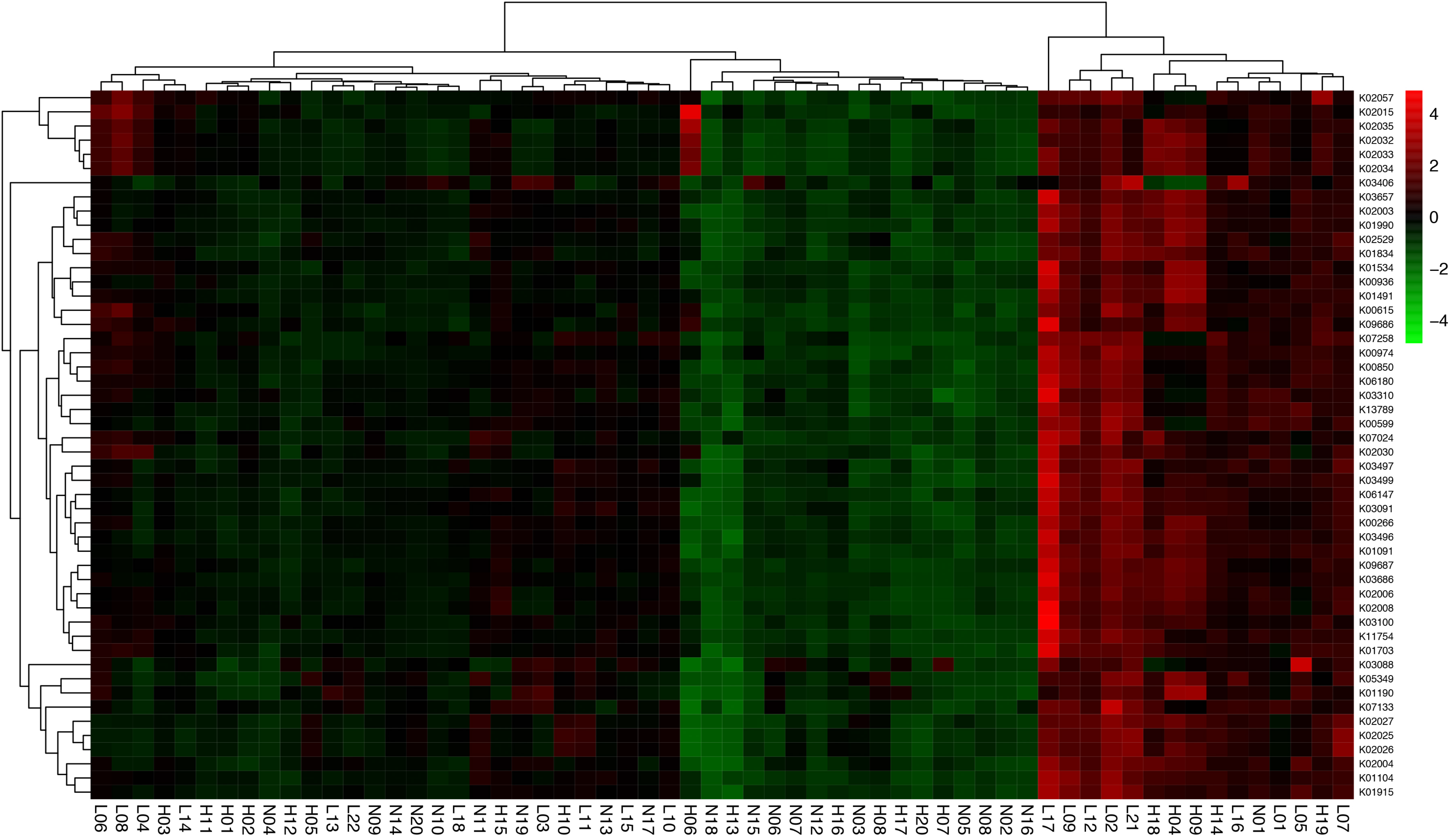

Figure S4 Heatmap for KEGG Orthologies distribution in samples from different groups

Heatmap for KEGG Orthologies distribution in stool samples of pregnant women in the control, mild ICP and severe ICP groups (clustered by both group, H stands for severe ICP group, L for mild ICP group and N for control group).
